# Why Has Deep Brain Stimulation Had So Little Impact in Psychiatry?

**DOI:** 10.3389/fneur.2021.757142

**Published:** 2021-12-14

**Authors:** Roel J. T. Mocking, Ilse Graat, Damiaan Denys

**Affiliations:** Department of Psychiatry, Amsterdam UMC, Location AMC, University of Amsterdam, Amsterdam, Netherlands

**Keywords:** deep brain stimulation, psychiatry, neurology, precision medicine, implementation gap

## Abstract

Over two decades ago, the first scientific publication on deep brain stimulation (DBS) in psychiatry was published. The evidence for effectiveness of DBS for several psychiatric disorders has been steadily accumulating since the first report of DBS for Obsessive Compulsive Disorder (OCD) in 1999. However, the number of psychiatric patients treated with DBS is lagging behind, particularly in comparison with neurology. The number of patients treated with DBS for psychiatric indications worldwide probably does not exceed 500, compared to almost 300,000 patients with neurological disorders that have been treated with DBS within the same period of 20 years. It is not the lack of patients, knowledge, technology, or efficacy of DBS that hinders its development and application in psychiatry. Here, we discuss the reasons for the gap between DBS in neurology and in psychiatry, which seemed to involve the scientific and social signature of psychiatry.

## Introduction

It has been roughly two decadess since deep brain stimulation (DBS) was first applied in psychiatry. A short letter to the Lancet in 1999 described the success of acute stimulation of the internal capsule in three out of four patients with obsessive-compulsive disorder (OCD) (1). What has then happened in the past 20 years with DBS in psychiatry?

Since 1999, the number of publications has increased to about 70 per year. Sham-controlled trials and meta-analyses have demonstrated effectiveness for DBS in OCD. Therefore, DBS has been recognized in some countries as an accepted treatment for OCD and has also been included in the guidelines regarding reimbursement by health insurance companies. Thanks to new technological developments, including sensing electrodes and the 7-tesla scans, clinicians are starting to understand the mechanisms of DBS for psychiatric disorders.

## Psychiatry vs. Neurology

Yet, if one takes a closer look at the development of DBS in OCD over the past 20 years, one will be struck by its slow progression. The large and growing number of published meta-analyses, policy documents, guidelines, and ethical protocols is in dire contrast compared to the scarcity of primary studies with patients. All in all, the number of patients with OCD treated with DBS is low. Based on the published studies and the number of research groups, we estimated that the number of DBS receiving patients with OCD worldwide probably does not exceed 300. These low numbers are especially poignant when compared to those in neurology. In the same 20 years, almost 300,000 patients with neurological disorders, such as Parkinson's Disease (PD), have been treated with DBS.

Why does DBS develop so smoothly in neurology and so laboriously in psychiatry? How can we understand the thousand-fold difference in application, although technology and treatment results are similar? In PD, the effect sizes for the reduction of motor symptoms and functional improvement are large (SMD > 0.80) (2), while in OCD, the effect size for reduction of OCD-symptoms is comparable (hedges' g = 2.5, 95%CI = 1.9–3.0) (3). Of the patients with OCD that showed insufficient response to pharmacotherapy or psychotherapy, more than half show complete response to DBS in randomized placebo-controlled trials (RCTs) (I^2^ = 0%; *p* = 0.003), with a number needed to treat of 3 (4). Experiences of individual patients are in line with these numbers (5). Side effects include perioperative complications and mostly transient hypomanic symptoms but do infrequently lead to drop-out (4), and are also generally comparable between neurology and psychiatry. Now, why are there so few patients treated with DBS in psychiatry despite the number of severe therapy-refractory patients and the mental burden it incurred is no less than movement disorders?

## Stagnation of DBS in Psychiatry

Needless to say, neurology and psychiatry are different. One may, for example, hypothesize that the stalled development of DBS in psychiatry is due to cost issues. In some countries, like in the US, the clinical follow-up period following the DBS procedure is not reimbursed, though several studies have shown that DBS is cost-effective and cost-saving in OCD (6, 7). A recent study from a group in the US reported that half of the patients who were found eligible for DBS by their physicians were not receiving DBS because insurance companies refused to pay for the treatment (8). However, in a country like the Netherlands, DBS for OCD is reimbursed for patients with refractory OCD, but the number of patients treated with OCD is still not as high as one would expect based on the prevalence of refractory OCD (9). Therefore, alternative explanations should be considered. Some clinicians preferred ablative surgery instead of DBS because DBS would be less effective than capsulotomy or cingulotomy. However, a recent meta-analysis showed that DBS is equally effective as ablative surgery in treatment refractory OCD (10). Others may prefer non-invasive neurostimulation techniques like transcranial magnetic stimulation (TMS) than DBS, but contrary to the latter, more than half of TMS studies found no significant benefit over sham stimulation in OCD (3). One may hypothesize that patients are more difficult to recruit, that companies are less interested, that funding agencies are less convinced, or that psychiatrists lacked sufficient expertise.

However, there seemed to be more fundamental problems that hamper DBS advancement in psychiatry: the distrust of psychiatrists in the neurobiology of psychiatric disorders, the social stigma of psychiatry, and the ethical concerns involved in mental disorders.

First, neurology and psychiatry differ profoundly in their relation to neurosurgery and neuroscience. Unlike neurology, psychiatry is a far more heterogeneous and an outspoken discipline. Some psychiatrists focus on its neurobiological perspective while others on its psychological or social dimension. The emphasis on one domain often goes along with denial of the other. For some patients and professionals, psychiatric disorders are not associated with brain dysfunction (11). Compared to motor effects of DBS in neurology, effects of DBS on psychiatric phenomena and experiences like in OCD may be seen as reductionistic and dehumanizing.

Second, psychiatry is still surrounded by social stigma. Public and self-stigma are considered to be two of the main barriers to adequate treatment for psychiatric disorders (12). For example, <2% of the global median of government health expenditure covers mental health, while mental illness is responsible for more than 10% of the total disease burden (13). Also, mental health research is underfunded relative to the burden of disease, which hampers the development and implementation of new treatments (14). Compared to other diseases (e.g., cancer), the relative amount of funding for mental health research is a factor of 25 lower, with less public contribution (15).

Third, raising funds for DBS research is often hampered by reviewers who find it unethical to treat a psychiatric patient with electrodes, maybe partly due to the negative historical influence of the anti-psychiatry movement and the past experiences with psychosurgery. Lobotomy and pre-modern electroconvulsive therapy are infamous examples of psychosurgery and brain stimulation that are extensively and ominously portrayed in popular media (16). However, modern DBS is a multidisciplinary treatment that is carried out in a regulated and controlled manner. Should we still let the past cast its shadow over our clinical practice today?

## Discussion

In summary, we hypothesize that lack of belief in the biology of psychiatric disorders, social stigma surrounding psychiatry, and ethical concerns hamper psychiatry in the development of DBS for severe and refractory disorders. If we want to give our patients a better chance, we need to invest in knowledge transfer. Education of colleagues, of students, and of people, who are working in government, research funding, and insurance companies, is pivotal to overcome stigma and ethical concerns. Further extension of the body of evidence by standardized trials and systematic follow-ups of OCD-DBS patients, including broad outcome measures, such as quality of life, may be needed to ensure that DBS can no longer be ignored as an optional treatment in psychiatry. In the end, DBS can only fulfill its potential impact if our society is capable to alter its attitude toward psychiatry for the next 20 years.

## Data Availability Statement

The original contributions presented in the study are included in the article/supplementary material, further inquiries can be directed to the corresponding author/s.

## Author Contributions

All authors listed have made a substantial, direct, and intellectual contribution to the work and approved it for publication.

## Funding

RM is supported by an ABC Talent Grant.

## Conflict of Interest

The authors declare that the research was conducted in the absence of any commercial or financial relationships that could be construed as a potential conflict of interest.

## Publisher's Note

All claims expressed in this article are solely those of the authors and do not necessarily represent those of their affiliated organizations, or those of the publisher, the editors and the reviewers. Any product that may be evaluated in this article, or claim that may be made by its manufacturer, is not guaranteed or endorsed by the publisher.

## References

[1] Nuttin B Cosyns P Demeulemeester H Gybels J Meyerson B. Electrical stimulation in anterior limbs of internal capsules in patients with obsessive-compulsive disorder. Lancet. (1999) 354:1526. 10.1016/S0140-6736(99)02376-4 10551504

[2] Perestelo-Pérez L Rivero-Santana A Pérez-Ramos J Serrano-Pérez P Panetta J Hilarion P. Deep brain stimulation in Parkinson's disease: meta-analysis of randomized controlled trials. J Neurol. (2014) 261:2051–2060. 10.1007/s00415-014-7254-6 24487826

[3] Bergfeld IO Dijkstra E Graat I de Koning P van den Boom BJG Arbab T. Invasive and non-invasive neurostimulation for OCD. Curr Top Behav Neurosci. (2021) 25, 359–377. 10.1007/7854_2020_206 33550567

[4] Peste Martinho F Silva Duarte G Simões do Couto F. Efficacy, effect on mood symptoms, and safety of deep brain stimulation in refractory obsessive-compulsive disorder: a systematic review and meta-analysis. J Clin Psychiatry. (2020) 81:19r12821 10.4088/JCP.19r12821 32459406

[5] de Haan S Rietveld E Stokhof M Denys D. Effects of deep brain stimulation on the lived experience of obsessive-compulsive disorder patients: in-depth interviews with 18 patients. PLoS One. (2015) 10:e0135524 10.1371/journal.pone.0135524 26312488 PMC4552296

[6] Moon W Kim SN Park S Paek SH Kwon JS. The cost-effectiveness of deep brain stimulation for patients with treatment-resistant obsessive-compulsive disorder. Medicine. (2017) 96:e7397. 10.1097/MD.0000000000007397 28682894 PMC5502167

[7] Ooms P Blankers M Figee M Bergfeld IO van den Munckhof P Schuurman PR. Cost-effectiveness of deep brain stimulation versus treatment as usual for obsessive-compulsive disorder. Brain Stimul. (2017) 10:836–842. 10.1016/j.brs.2017.04.120 28457837

[8] Pinckard-Dover H Ward H Foote KD. The decline of deep brain stimulation for obsessive–compulsive disorder following FDA humanitarian device exemption approval. Front Surg. (2021) 8:44. 10.3389/fsurg.2021.642503 33777998 PMC7994854

[9] Denys D Graat I Mocking R de Koning P Vulink N Figee M. Efficacy of deep brain stimulation of the ventral anterior limb of the internal capsule for refractory obsessive-compulsive disorder: a clinical cohort of 70 patients. Am J Psychiatry. (2020) 177:265–271. 10.1176/appi.ajp.2019.19060656 31906709

[10] Hageman SB van Rooijen G Bergfeld IO Schirmbeck F de Koning P Schuurman PR. Deep brain stimulation versus ablative surgery for treatment-refractory obsessive-compulsive disorder: a meta-analysis. Acta Psychiatr Scand. (2021) 143, 307–308. 10.1111/acps.13276 33492682

[11] Chung JY Insel TR. Mind the gap: neuroscience literacy and the next generation of psychiatrists. Acad psychiatry J Am Assoc Dir Psychiatr Resid Train Assoc Acad Psychiatry. (2014) 38:121–3. 10.1007/s40596-014-0054-6 24619911

[12] Corrigan P. How stigma interferes with mental health care. Am Psychol. (2004) 59:614–25. 10.1037/0003-066X.59.7.614 15491256

[13] WHO/UNDP. Making the Investment Case for Mental Health: A WHO / UNDP Methodological Guidance Note. Geneva: World Health Organization (2019)

[14] Woelbert E Lundell-Smith K White R Kemmer D. Accounting for mental health research funding: developing a quantitative baseline of global investments. Lancet Psychiatry. (2021) 8:250–8. 10.1016/S2215-0366(20)30469-7 33242400

[15] MQ: Transforming Mental Health. UK Mental Health Research Funding 2014–2017. (2017). Available online at: https://www.mqmentalhealth.org/wp-content/uploads/UKMentalHealthResearchFunding2014-2017digital.pdf

[16] Caruso JP Sheehan JP. Psychosurgery, ethics, and media: a history of walter freeman and the lobotomy. Neurosurg Focus. (2017) 43:E6. 10.3171/2017.6.FOCUS17257 28859561

